# Paracrine Effects of Bone Marrow Soup Restore Organ Function, Regeneration, and Repair in Salivary Glands Damaged by Irradiation

**DOI:** 10.1371/journal.pone.0061632

**Published:** 2013-04-24

**Authors:** Simon D. Tran, Younan Liu, Dengsheng Xia, Ola M. Maria, Saeed Khalili, Renee Wan-Jou Wang, Vu-Hung Quan, Shen Hu, Jan Seuntjens

**Affiliations:** 1 Faculty of Dentistry, McGill University, Montreal, Quebec City, Canada; 2 Centre Hospitalier de l’Université de Montréal, Montreal, Quebec City, Canada; 3 School of Dentistry, University of California Los Angeles, Los Angeles, California, United States of America; 4 Department of Oncology, Medical Physics Unit, McGill University, Montreal, Quebec City, Canada; National Institutes of Health, United States of America

## Abstract

**Background:**

There are reports that bone marrow cell (BM) transplants repaired irradiated salivary glands (SGs) and re-established saliva secretion. However, the mechanisms of action behind these reports have not been elucidated.

**Methods:**

To test if a paracrine mechanism was the main effect behind this reported improvement in salivary organ function, whole BM cells were lysed and its soluble intracellular contents (termed as “BM Soup”) injected into mice with irradiation-injured SGs. The hypothesis was that BM Soup would protect salivary cells, increase tissue neovascularization, function, and regeneration. Two minor aims were also tested a) comparing two routes of delivering *BM Soup*, intravenous (I.V.) versus intra-glandular injections, and b) comparing the age of the *BM Soup’s* donors. The treatment-comparison group consisted of irradiated mice receiving injections of living whole BM cells. Control mice received irradiation and injections of saline or sham-irradiation. All mice were followed for 8 weeks post-irradiation.

**Results:**

*BM Soup* restored salivary flow rates to normal levels, protected salivary acinar, ductal, myoepithelial, and progenitor cells, increased cell proliferation and blood vessels, and up-regulated expression of tissue remodeling/repair/regenerative genes (MMP2, CyclinD1, BMP7, EGF, NGF). *BM Soup* was as an efficient therapeutic agent as injections of live BM cells. Both intra-glandular or I.V. injections of *BM Soup*, and from both young and older mouse donors were as effective in repairing irradiated SGs. The intra-glandular route reduced injection frequency/dosage by four-fold.

**Conclusion:**

*BM Soup*, which contains only the cell by-products, can be advantageously used to repair irradiation-damaged SGs rather than transplanting whole live BM cells which carry the risk of differentiating into unwanted/tumorigenic cell types in SGs.

## Introduction

There are an estimated 40,000 new cases of oro-pharyngeal cancer and 8,000 estimated deaths due to this disease each year in the United States [1]. Irradiation (IR) is a key component of therapy for these patients, but this causes damage to the salivary glands (SGs), particularly the acinar cells [2]. These cells are the principal site of fluid secretion (saliva) and such patients experience considerable morbidity and discomfort, ranging from symptoms such as xerostomia, dysphagia, dental caries, altered taste, oro-pharyngeal infections and pain [3]. In many patients, all the salivary secretory tissue is lost, which means no suitable treatment is available since existing pharmacologic therapy depends on stimulating residual acinar cells.

Studies of adult stem cell-based therapy have reported an overall improvement of (non-hematologic) organ function. The proposed mechanisms by which bone marrow (BM) cells improve organ functions have been investigated for the past 10–15 years. Initial reports proposed the ability of BM cells to (trans)differentiate into cells of a non-marrow/non-hematopoietic lineage (such as the brain, heart) was the mechanism of action for tissue regeneration [4], [5]. Then reports on fusion of BM cells with parenchymal cells of other tissues were documented [6], [7]. The third mechanism proposed was vasculogenesis from endothelial progenitor cells contained within peripheral blood stem cells or from BM mononuclear cells [8]. Lastly, the most recent mechanism of action is that BM cells provide a local paracrine effect [9], [10], [11]. These four mechanisms of action by BM cells have been thoroughly discussed in recent reviews [12], [13]. In summary, tissue and vascular regeneration have been initially proposed as mechanisms of stem cell action [10], [11], [14]. However at closer investigation, the low frequency of stem cell engraftment or the low number of newly generated parenchymal and vascular cells (either by cell transdifferentiation or fusion) could not fully explain organ improvement [10]. Accordingly, several authors have proposed an alternative paradigm that transplanted stem cells release soluble factors that, acting in a paracrine fashion, contribute to organ repair and regeneration. This hypothesis suggests that cytokines, growth and other factors induce cytoprotection, neovascularization, and mediate endogenous tissue regeneration via activation of resident tissue stem cells. In addition, tissue remodeling and organ function is affected by these paracrine factors [10].

Recent findings from our group demonstrated BM cell transplants repaired SGs and re-established saliva secretion in mice that received IR to their neck area [15]. This current paper aims to test if a paracrine mechanism is the main cause behind the reported improvement of salivary organ function. To test this, whole BM cells will be lysed and its soluble intracellular contents injected into mice with IR-injured SGs. A former group of researchers has coined this product as “bone marrow cell extract” [16], but this paper will rename it as *“Bone Marrow Soup” (BM Soup)* to first remind the readers that this product is made of a mixture of unknown soluble factors, and second, as a historical analogy to *Chicken Soup* (reported with medicinal properties, but its specific composition/ingredients are still not known) [17], [18], [19].

The first aim of this study is to test the efficiency of *BM Soup* and investigate its mechanisms of action in repairing SGs of mice damaged by IR. The hypothesis is that *BM Soup* protects salivary cells, increases tissue neovascularization, function, and regeneration. In addition, two minor aims are tested: a) comparing two routes of delivering *BM Soup* to SGs (I.V. versus intra-glandular injection), and b) comparing the age of the *BM Soup’s* donors.

## Results

### *BM Soup* Restored Salivary Organ Function, Protected Cells, Increased Proliferation of Cells and Blood Vessels, and up-regulated Expression of Tissue Repair/regenerative Genes

**Figure 1:**
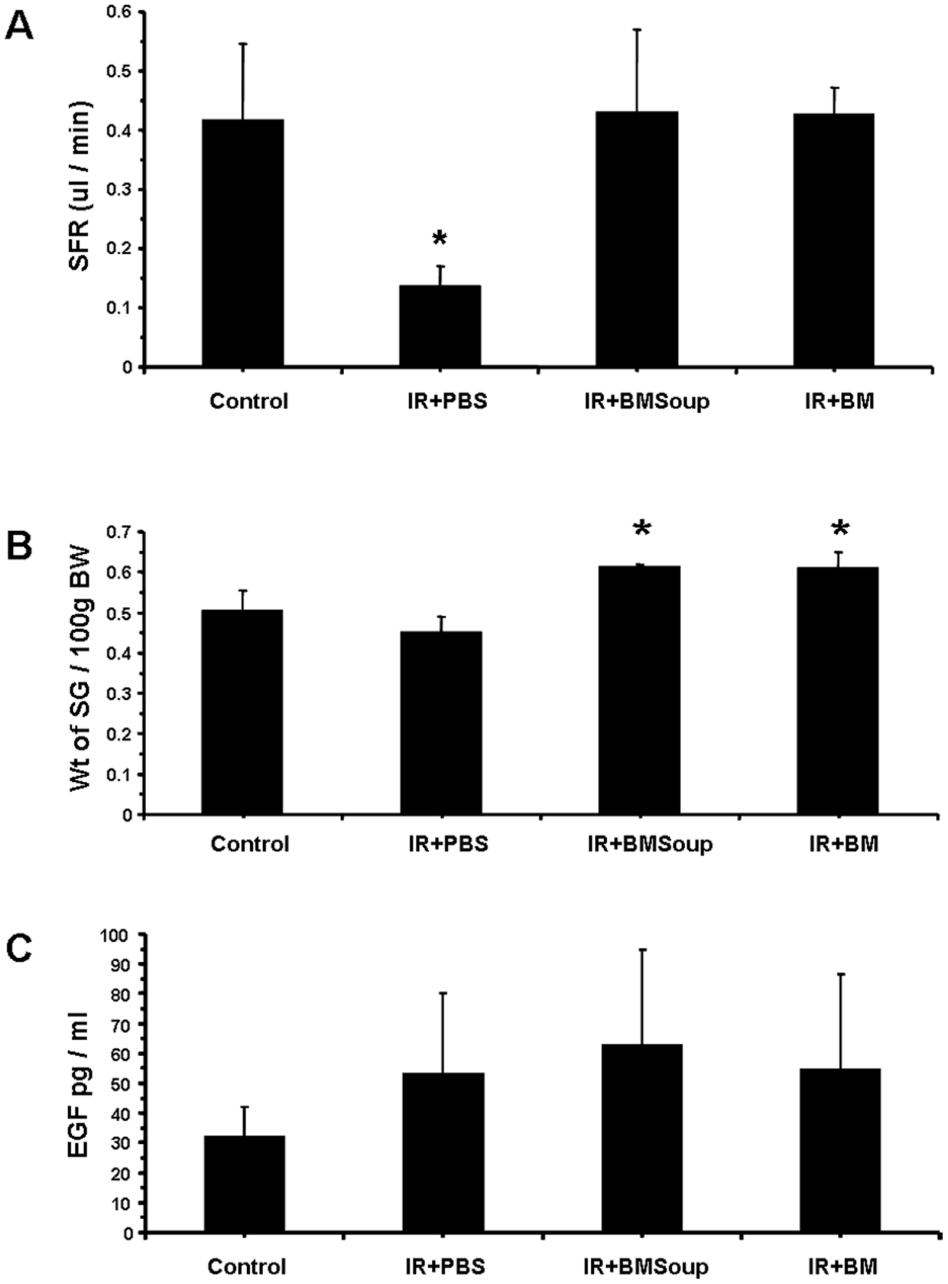
*Bone Marrow Soup* re-established saliva secretion following irradiation. **A.** At week 8 post-irradiation, BM Soup-treated mice (*IR+BMSoup*) had their salivary flow rate (SFR; ul/min/per g of body weight) restored to normal levels (100%; 0.4 ul/min) when compared to sham-irradiated mice (*Control*) and those transplanted with whole live BM cells (*IR+BM*). Mice receiving an intravenous injection of PBS (*IR+PBS*; vehicle control group) had 60% reduction in SFR (* P<0.05). **B.** Weights of the salivary glands relative to 100g of their body weights were higher in *IR+BMSoup* and *IR+BM* mice (* P<0.05). **C.** Saliva levels of epidermal growth factor (EGF) were not statistically different among the four groups of mice (P>0.05). N = 5 to 9 mice per group.

The first part of this study assessed the efficacy of *BM Soup* in repairing SGs that were damaged by IR injury. At week 8 post-IR, *BM Soup*-treated mice (*IR+BMSoup*) had their salivary flow rate (SFR) restored to normal levels (100%) while PBS-treated mice (*IR+PBS*) had a 60% reduction (Fig. 1A, p<0.05). SFR of *BM Soup*-treated mice were comparable to that of mice in the Control or BM-treated group (*IR+BM*). These data indicated that *BM Soup* treatment was superior to no treatment (IR+PBS injection) in re-establishing saliva secretion. Weights of the SGs (relative to their body weights) were higher in *IR+BM Soup* mice when compared to *IR+PBS* mice (Fig. 1B, p<0.05). All mouse groups had comparable concentrations of salivary EGF (Fig 1C).

**Figure 2:**
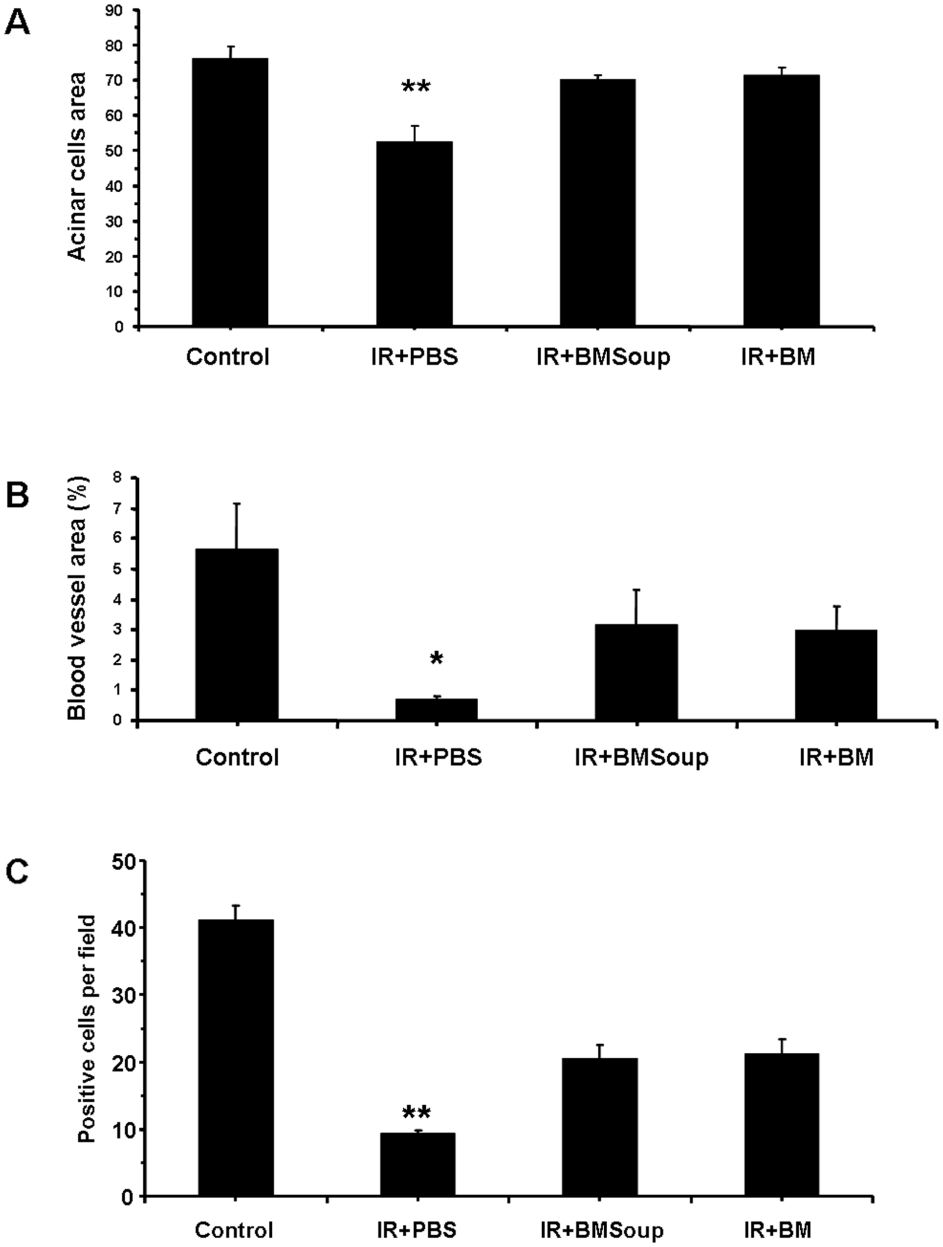
Role of *Bone Marrow Soup* on acinar cells, blood vessels and cell proliferation rate. **A**. Area in salivary gland occupied by acinar cells (quantification of 10 representative fields per gland/mouse with NIH image J software). *BMSoup*-treated group had a comparable percentage of surface area occupied by acinar cells as Control (sham-irradiated) and IR+BM groups, but a higher surface area with acinar cells than irradiated mice (IR+PBS) (*P<0.05, **P<0.01; N = 5 mice per group). **B.** *BMSoup*-treated mice had higher density of blood vessels (by von Willebrand factor) than irradiated mice. **C.** *BMSoup*-treated mice had higher cell proliferation (by PCNA) than irradiated mice.

**Figure 3:**
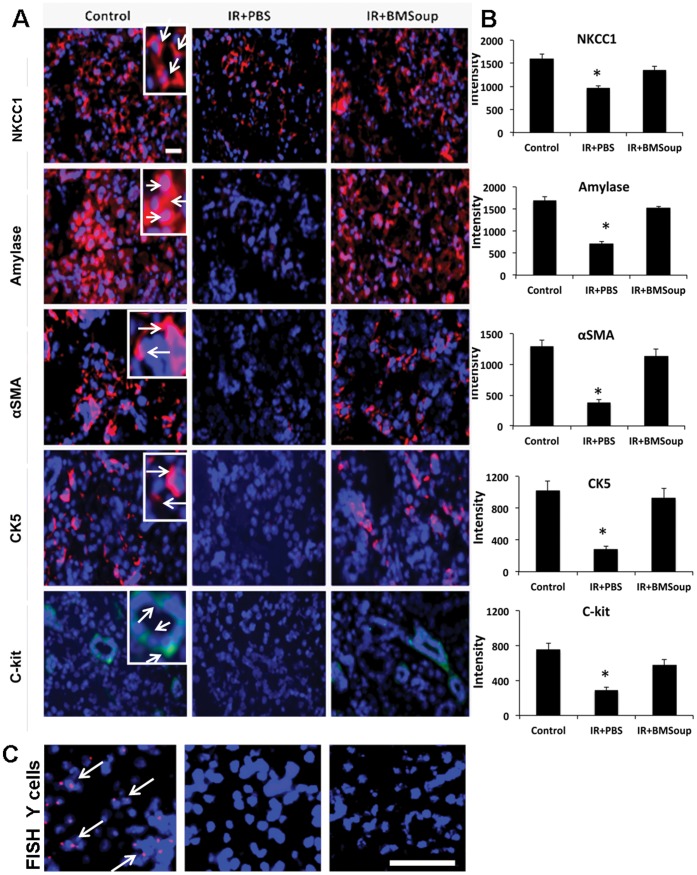
Role of *Bone Marrow Soup* in cytoprotection. **A.** Immunofluorescence staining of specific subpopulations of cells in submandibular glands at 8 wks post-irradiation. Cell markers are shown in red (or green for c-kit); cell nuclei are in blue. *BM Soup-*treated mice had a higher number of cells positive for NKCC1 and amylase (used here as markers of acinar cells; see white arrows in inset of a magnified region), smooth muscle actin (marker for myoepithlial cells), CK5 and c-kit (markers for ductal and salivary progenitor cells) than PBS-treated mice. All photographs were taken at the same magnification; scale bar (white) is 220 um; inset shows a higher magnification of selected cells. **B.** Quantification of protein fluorescence (intensity) expressed in 3–5 fields of 400 um^2^ area/per gland by Volocity software (N = 5 mice per group). *BMSoup* shows comparable proportions of acinar, ductal, myoepithelial, and stem cells as non-irradiated control/healthy mice. IR+PBS group has less protein intensity when compared to *Control* or *BMSoup*-treated mice (*P<0.05). **C.** Fluorescence in situ hybridization using a Y-chromosomal probe. Left picture with “red dots” localize (expected) male cells in a male mouse salivary gland (positive control). White arrows are pointing to some male cells. Cell nuclei are in blue. Middle picture was taken from a female mouse (negative control; no red cells are seen). Right picture confirmed that *BM Soup* did not contain any Y-chromosome (male) transplanted cells in female irradiated salivary tissues. White scale bar is 38 um.

*BM Soup* was found to protect several cell populations in irradiated-SGs. Alcian blue and periodic acid-Schiff (PAS) staining revealed that the percentage of surface area occupied by acinar cells was comparable in *Control*, *IR+BMSoup* and *IR+BM* mice, while significantly increased when compared to IR+PBS treated mice (Fig. 2A, p<0.05). Von Willebrand factor staining showed that the number of blood vessels was markedly decreased (6-fold less) in IR+PBS mice when compared to non-IR control mice, but that *BM Soup* or BM cells treatment allowed (4-fold) more blood vessels to proliferate (Fig. 2B, p<0.05). *BM Soup* was found to favor tissue repair and remodeling. Cell proliferation (PCNA) was 2.5-fold higher in *BM Soup*-treated mice than IR+PBS mice (Fig. 2C, p<0.05). Immunostaining was used to localize and quantify specific subpopulations of cells. *BM Soup-*treated mice had a higher number of cells positive for amylase and NKCC1 (markers of acinar cells), smooth muscle actin (marker for myoepithlial cells), CK5 and c-kit (markers for ductal and salivary progenitor cells) than PBS-treated mice (Fig. 3A–B, p<0.05). To guarantee that *BM Soup* contained no live or whole cells, we examined under the microscope for any remaining cells and nuclei using trypan blue or Hoescht33342 dye (Invitrogen). None was observed (data not shown). Also, since *BM Soup* was obtained from male mouse donors, we subsequently used a Y-chromosomal probe and fluorescence in situ hybridization (FISH) to detect any male cells in SGs of female C3H mice. No male cells were observed (Fig. 3C). These experiments confirmed that the solution of *BM Soup* did not contain any cells.

**Figure 4:**
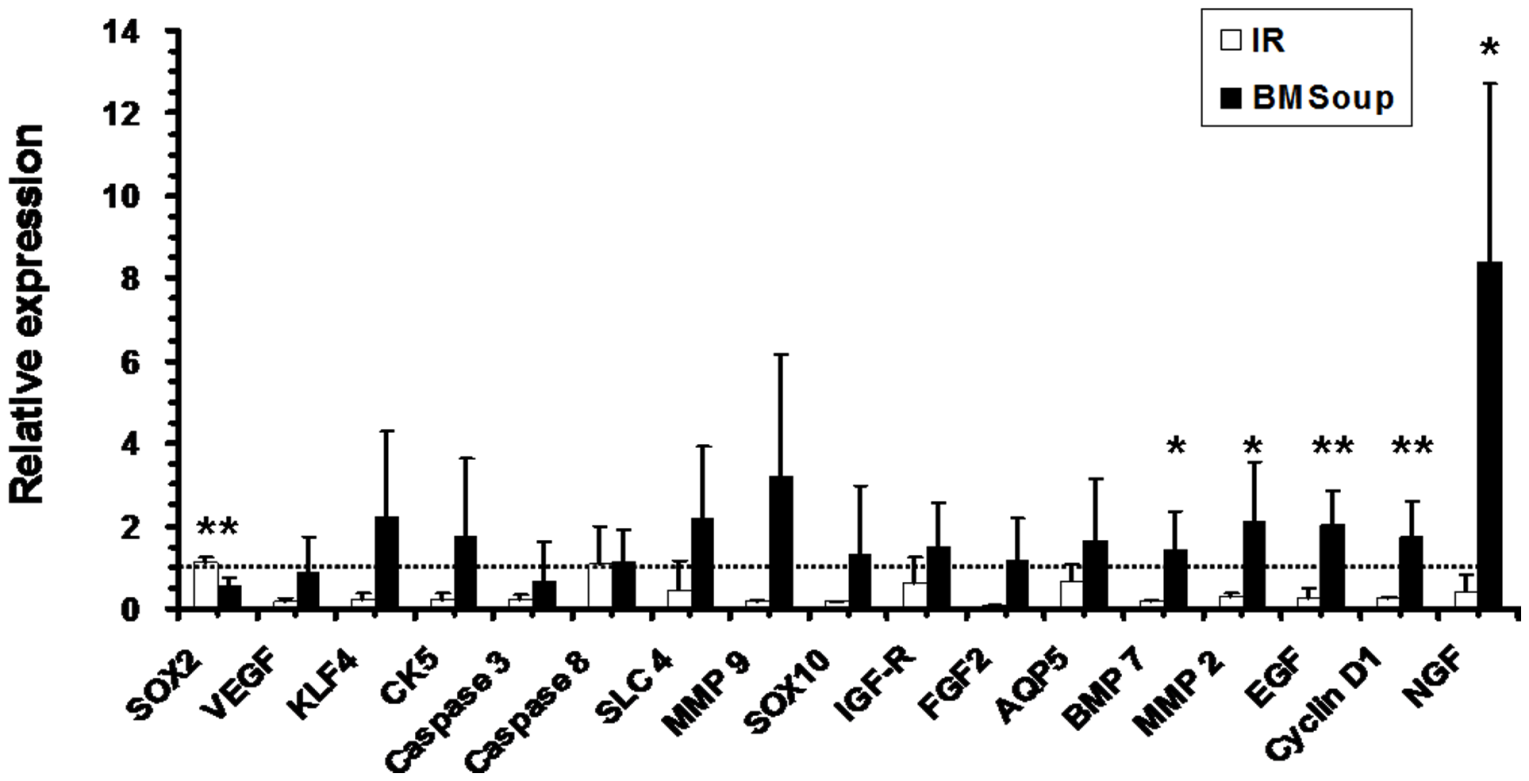
Relative gene expression of key factors involved in salivary gland development, repair and regeneration. qRT-PCR results at 8-week post-irradiation between Control, Irradiated (IR only) and BMSoup-treated mice. mRNAs were significantly up-regulated in *BM Soup* versus IR mice for MMP2 (an enzyme in tissue breakdown), BMP7, EGF, Cyclin D1 (cell cycle G1/Synthesis) and NGF (*P<0.05, **P<0.01; N = 5 mice per group). There was a downregulation of Sox2 (transcription factor in stem cell renewal). Y-axis shows the relative expression of the gene compared to GAPDH. Horizontal dashed line represents the relative gene expression level of 1 in mice from the Control group (sham-irradiated mice).

A panel of genes important to SG development, tissue regeneration and repair was examined in *BM Soup*-treated versus PBS-treated mice. In general, all genes surveyed for this study were up-regulated (Fig 4). Specifically, MMP2 (an enzyme in tissue breakdown), Cyclin D1 (cell cycle G1/Synthesis), and three growth factors involved in salivary gland development/maintenance (BMP7, EGF, NGF) were significantly up-regulated in *IR+BM Soup* versus IR+PBS mice (Fig 4; p<0.05), while only Sox2 (a transcription factor in stem cell renewal) was down-regulated. Taken together, all these above data (from Figs 1–4) indicated the efficacy of *BM Soup* in repairing IR-damaged SGs.

### Both Intra-glandular and Intravenous Injection Methods were Effective in Delivering *BM Soup* for the Tissue Repair and Restoration of Salivary Organ Function

**Figure 5:**
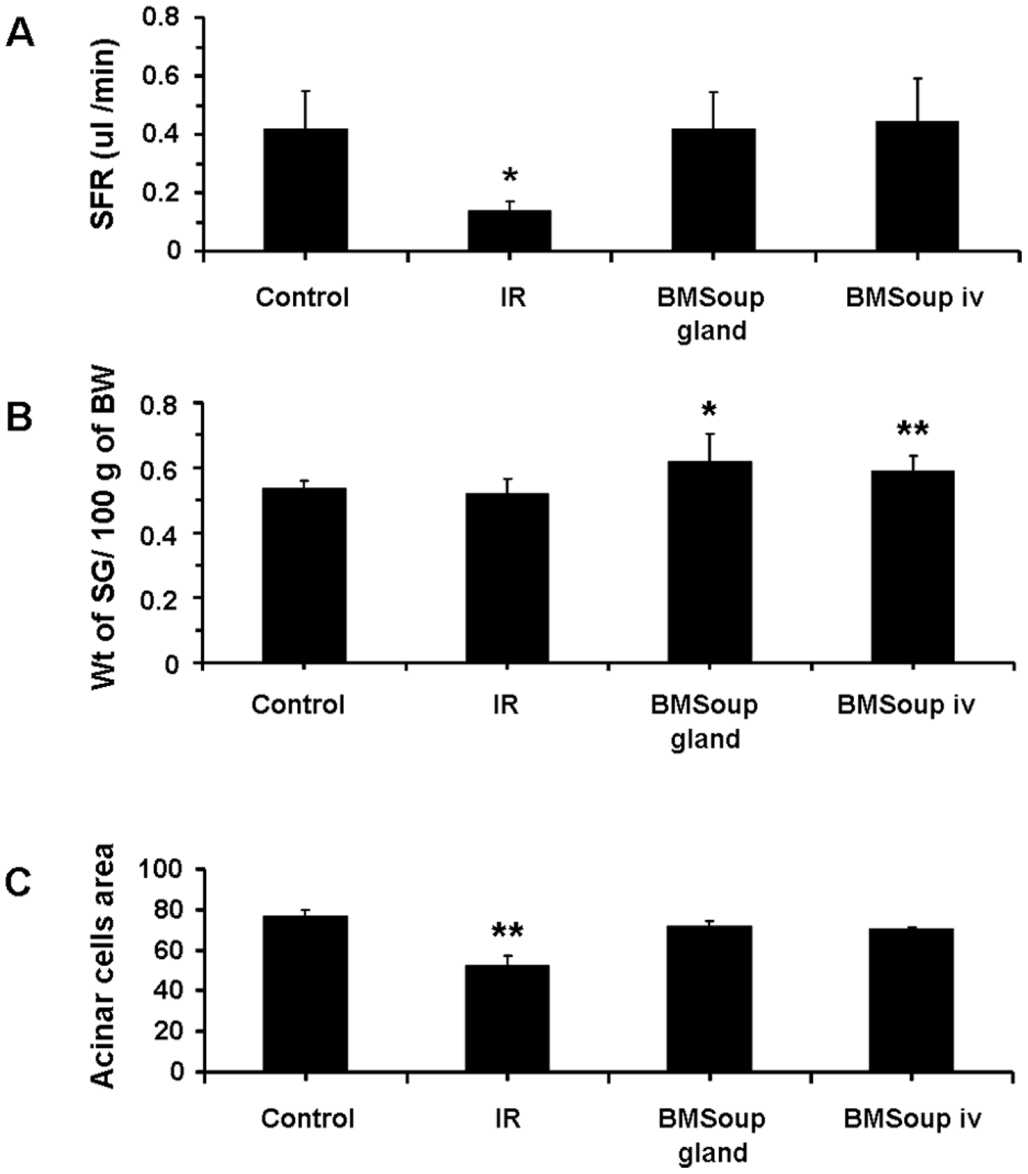
Intra-glandular and intravenous injections of *BM Soup* were both effective for restoration of salivary organ function. A. At week 8 post-irradiation, mice that were injected with BM Soup, either intra- glandularly (*BMSoup gland*) or intra-venously (*BMSoup iv*), had their salivary flow rate restored to normal levels (100%; 0.4 ul/min) when compared to sham-irradiated mice (*Control*). Mice that were irradiated and not treated (*IR* group) had 60% reduction in SFR (*P<0.05, **P<0.01; N = 5 to 9 mice per group). B. Weights of the salivary glands were higher in *BMSoup*-treated mice, either by intra-glandular or by IV injection. C. Both intra-glandular and IV delivered *BMSoup*-treated mice had a comparable percentage of surface area occupied by acinar cells as Control (sham-irradiated) mice. Irradiated mice injected with PBS had significantly a lower percentage of surface area with acinar cells.

**Figure 6:**
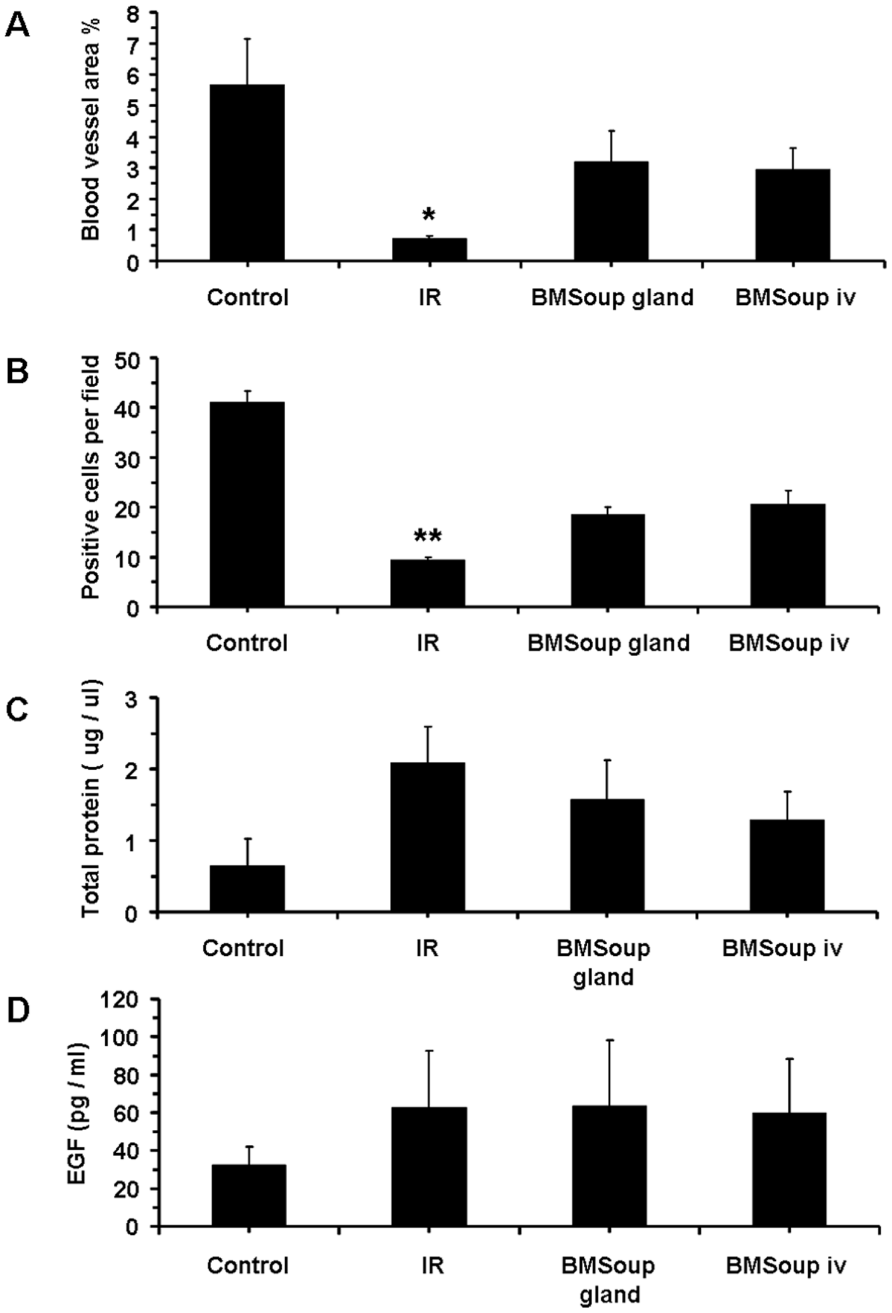
Intra-glandular and intravenous injections of *BM Soup* were both effective for tissue repair. **A and B.** Both intra-glandular and IV *BMSoup*-treated mice had higher density of blood vessels (by von Willebrand factor) and cell proliferation (by PCNA) than irradiated mice. **C and D.** Saliva levels of total proteins and epidermal growth factor (EGF) were not statistically different among the four groups of mice (P>0.05; N = 5 to 9 mice per group).

**Figure 7:**
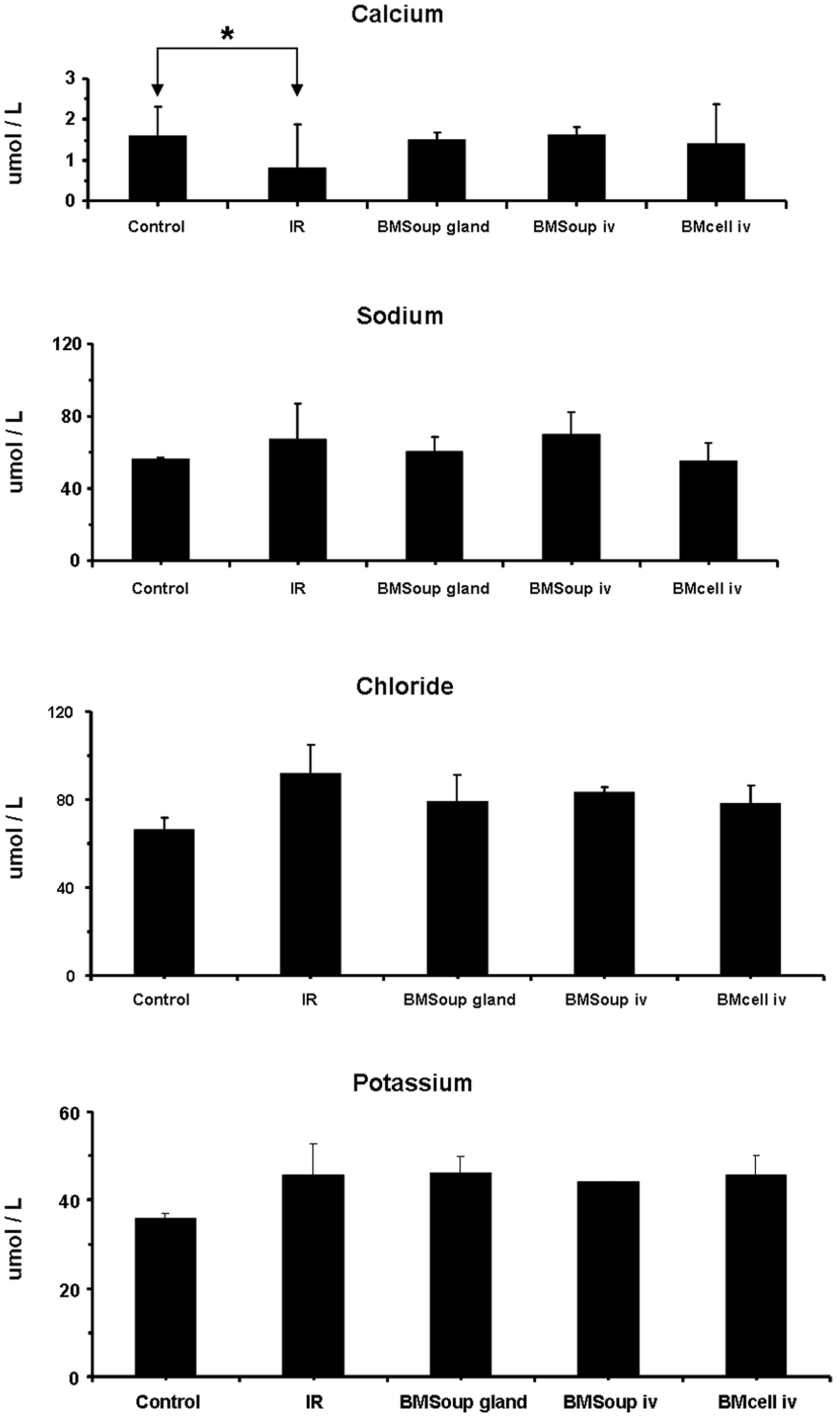
Levels of key electrolytes in saliva. Electrolytes in saliva (such as sodium, potassium, chloride, and calcium concentration levels) were not different among groups, except for calcium levels (P>0.05; N = 5 to 9 mice per group).

The second part of this study evaluated a systemic (I.V.) versus a local delivery route (intra-glandular injection) for *BM Soup* to repair SGs that were damaged by IR. Results at week 8 post-IR showed that both groups of mice treated with I.V. and intra-glandular injections of *BM Soup* had comparable SFRs, gland weights, cell proliferation rate, amount of acinar cells and blood vessels (Figs 5–6). These above mentioned measures were all lowered in IR+PBS mice (Figs 5–6; p<0.05). The composition (quality) of saliva was assessed and all mouse groups had comparable concentrations of salivary EGF, total proteins, and key electrolytes (sodium, potassium, chloride) (Figs 6–7). However, the levels of calcium was lower in *IR+PBS* mice (Fig 7, p<0.05). Overall, these data indicated that injections of *BM Soup* directly in the irradiated submandibular glands of C3H mice repaired the glands as efficiently as I.V. injections. One notable advantage for the intra-glandular delivery route was a lower frequency/number of injections. Only one intra-glandular injection was needed for the entire study as compared to four I.V. injections (twice a week for two consecutive weeks).

### *BM Soup* of both Young and Older Mouse Donors was Equally Effective in Repairing Irradiated SGs

**Figure 8:**
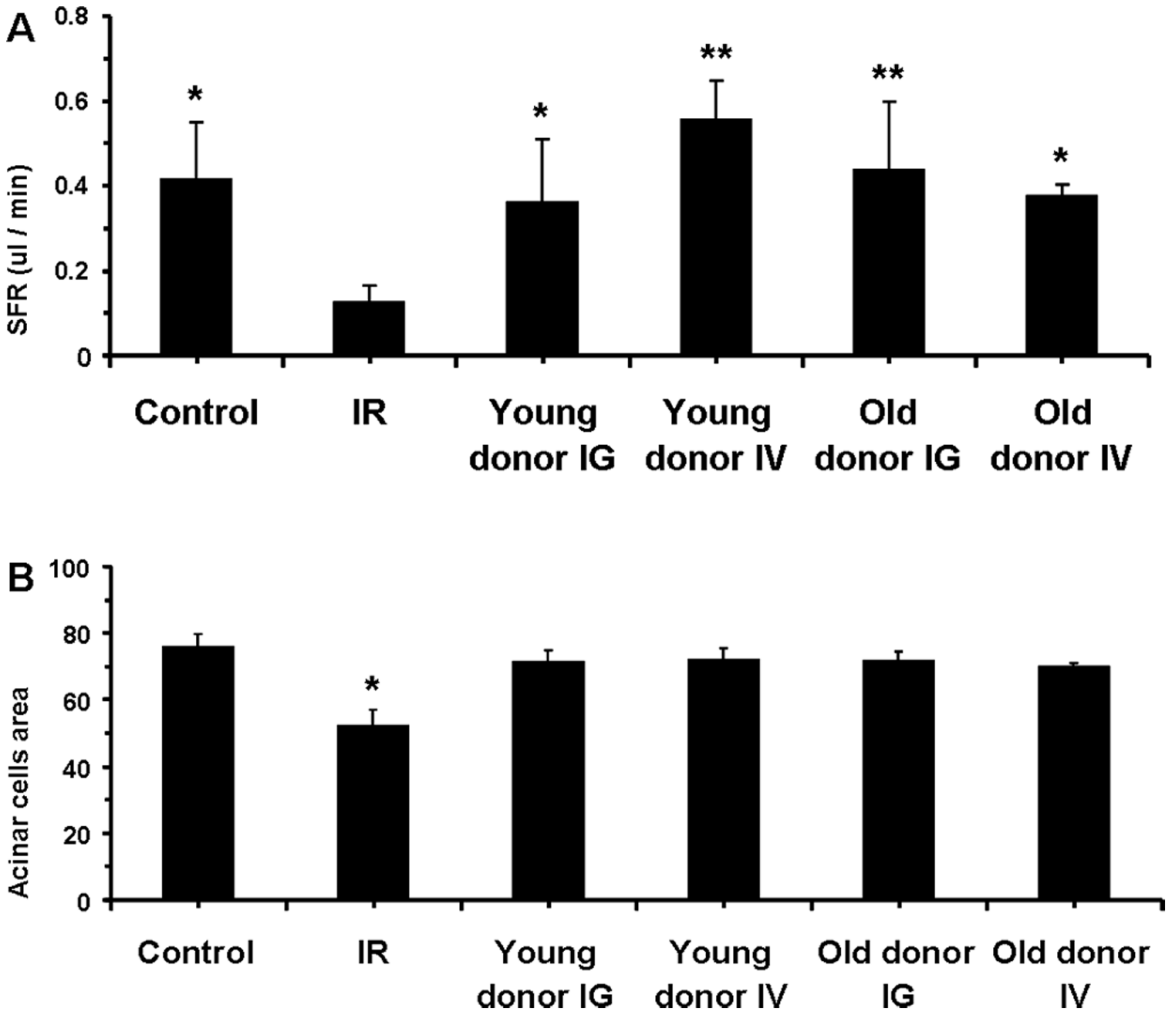
BM Soup of both young and older mouse donors was as effective in repairing irradiated salivary glands. **A.** At week 8 post-irradiation, salivary flow rate (SFR) of groups treated with *BM Soup* from both young (7–8 wks old) and older mouse donors (20–22 wks old) were comparable to sham-irradiated mice (*Control*) while the untreated mice (*IR* group) had 60% reduction in SFR. Both delivery routes for *BM Soup* (IG, intra-glandular; IV, intra-venous) were equally successful in re-establishing SFR to irradiated mice. **B.** The percentage of tissue surface area occupied by acinar cells was comparable between mice receiving *BM Soup* harvested from young and older donors, and from either an IV or IG injection delivery route. All mice treated with *BM Soup* had significantly a higher percentage of surface area with acinar cells than irradiated untreated mice (*IR* group). (*P<0.05, **P<0.01; N = 5 mice per group).

Reports from adult stem cell-based therapies indicated that more stem/progenitor cells could be isolated from younger donors [20], [21], [22], [23], [24]. Therefore, the third part of our study evaluated if *BM Soup* therapy success could be affected by the age of the donor mouse. Our data indicated that *BM Soup* harvested from young (7–8 wks old) and older mice donors (20–22 wks old) were both effective in repairing irradiated SGs. Both SFR and number of acinar cells were comparable between mice receiving *BM Soup* harvested from young and older donors, and from either an IV or intra-glandular injection delivery route (Fig 8). These findings suggest that *BM Soup* can be harvested from both young and adult mice, and that the delivery of *BM Soup* by IV or intra-glandular injection is equally efficient in repairing irradiated SGs.

## Discussion

The findings of this study were that: 1) *BM Soup* was as effective as whole live BM cells in repairing irradiated-SG, suggesting a paracrine effect of BM cells on SGs; 2) *BM Soup* restored salivary organ function, protected cells, increased proliferation of cells and blood vessels, and up-regulated expression of tissue repair/regenerative genes; 3) Intra-glandular or intravenous delivery of *BM Soup* was both effective for tissue repair and restoration of salivary organ function; and 4) *BM Soup* of both young and older mouse donors was effective in repairing irradiated SGs.

For functional restoration of damaged SGs, three approaches are being tested. The first approach is gene therapy and a recent successful trial restoring function in IR damaged human SGs has been reported [25]. The second approach is building an artificial SG using tissue engineering [26], [27]. The third approach is using (stem) cell-based therapy. Currently, stem/progenitor cells from two different organs are being investigated in mice: a) from the SG itself [28], [29], [30], [31], [32], [33] or b) from the BM [15], [34], [35], [36], [37], [38]. There is a general agreement, although with still a paucity of studies, that transplanted BM cells improved SG function in mouse models; while in humans, organ function has not been examined yet [13], [39], [40]. Both injected and mobilized BM cells (such as with granulocyte-colony stimulating factor) were found effective in partially restoring function in irradiated-SGs. Injections or vector-mediated transfer of growth factors (such as FGF2, IGF, KGF or VEGF) without cell transplants were also reported partially effective in repairing irradiated-SGs [41], [42], [43], [44], [45]. Taken these studies together, the data suggest that BM cells, specific growth factors, or combining both approaches could be used to restore SG organ function. This paper proposes an additional approach by delivering the soluble contents of lysed BM cells to SGs.

This paper highlights the importance of the paracrine effects of BM cell contents on irradiated-SG, and that intact BM cells may not be necessary. Proof-of-concept of paracrine cross-talking between SG cells and other cell populations (such as MSC or amniotic epithelial cells) was demonstrated by using a culture system that physically separated the cell populations [46], [47]. These findings on the paracrine effects of MSC versus SG cells are in agreement with studies from other organs. Particularly to those in cardiology where several studies on the paracrine effects of MSC (isolated from the BM) have shown that organ repair was due to the secretion of cytokines, chemokines, and growth factors [48]. The demonstration that injecting conditioned medium from MSC cultures exerted cardiomyocyte protection and improved cardiac function in mouse infarcted hearts provided an elegant proof of concept [49], [50]. However, delivering conditioned media would be impractical clinically. Therefore a group of cardiologists used a clinically-relevant and simple approach for delivering paracrine secretions of BM cells [16]. They hypothesized that if a paracrine mechanism was at play, use of the cell extract derived from whole BM (we use the term *BM Soup*) would enable them to detect the effects of any likely protein or factor that would be released. This cell extract was obtained by lysing BM cells, removing the insoluble material, and keeping only the intracellular soluble material. Living whole BM cells versus its cell extract were injected into mice which had surgically-induced myocardial infarction. Results showed similar improvement of cardiac function and a smaller infarct size in both mouse groups. This approach has many advantages (described below), in addition to being simple and likely to be translated to a clinical setting. Using *BM Soup* does not require the injection of live (stem) cells, which carry the risk of differentiating into unwanted/tumorigenic cell types in SGs. *BM Soup* includes all cell types of whole BM and consequently numerous proteins, cytokines and paracrine factors [16]. *BM Soup* is quicker to obtain than the use of a cell-derived conditioned media, which needs to be grown in culture and thus requires more time. *BM Soup* is not patient-specific. Recently, *BM Soup* from human origin was reported to improve cardiac function in a mouse model of myocardial infarction, without the need for immuno-compromised animals or antibiotic therapy [51]. Thus, eliminating the issues of cell rejection for allogeneic cells and negating the need for invasive BM harvesting for autologous cell transplantation could be additional advantages for *BM Soup* [51]. Overall, *BM Soup* can be obtained from an accessible cell source (i.e. bone marrow). There is potentially less need for histocompatible donors, immune suppression drugs, or immune rejection. Also there is no need to differentiate stem cells, if any, into the correct cell lineage desired and less tumorigenicity risk is associated because there are no live cells.

Salivary flow rate (SFR) directly reflects function of the glands and its decrease is the major clinical finding in patients with dry mouth following head and neck IR. Therefore SFR was the critical quantitative measure used throughout this study and *BM Soup* was found efficient in restoring salivary function (Figs 1, 5, 8). In addition, we complemented salivary flow data with measurements for proteins (total proteins), salivary electrolytes (Na, Cl, K, Ca), and a growth factor (EGF). These measurements on the composition (quality) of saliva were used as indicators of acinar or ductal cell function [52]. Most proteins secreted into saliva come from acinar cells and measuring the total protein concentration allowed functional assessment of the secretory status of acinar cells [52]. EGF is mainly produced in SGs and this study measured EGF in saliva as a way to assess the functional secretory status of the granular ductal cells. Electrolytes were measured because most NaCl is reabsorbed while potassium is secreted by ductal cells; these levels can be used as an indicator of ductal cell function [52]. Calcium was used as an indicator of acinar cell function (exocrine protein packaging). All measurements on the composition of saliva, used in this study, did not show a statistically significant difference between the control, IR, and treated mice (Figs 6–7). Only the salivary calcium concentration in non-treated irradiated mice was found lower than the non-irradiated control mice. This suggests a reduced function (or number) of acinar cells in irradiated mice.

Testing different routes of delivering therapeutic agents is of critical importance in treating a disease. For SG damaged by IR, an I.V. delivery can provide cytoprotection to endothelial cells, which affect the survival of acinar cells [42], [53]. On the other hand, a local delivery can provide higher levels of *BM Soup* in the gland, which is the main site of tissue injury (acinar cells), as well as reducing any systemic adverse side-effects of the therapeutic agent, if any. Our study unexpectedly found that, one time, injections of *BM Soup* in submandibular glands were as effective in restoring SG function as four I.V. injections through the tail vein (see Materials and Methods section). Therefore the intra-glandular delivery route is less time-consuming and only required a quarter of the *BM Soup* dosage. Clinically, we envision two feasible methods to deliver *BM Soup* to SGs. First is by intra-glandular injections. Such an approach is currently used to deliver botulinum toxin to reduce drooling [54]. Second is by cannulation of the SG duct and retrograde infusion, such as currently used in sialoscopy/sialography [55]. Retrograde infusion and cannulation is also being used in research to transfer genes to SG of IR patients [25]. The majority of cells in SG exist as a monolayer and cannulation allows the delivered therapeutic agent or viral vector direct access to all SG cells. Our group and collaborators have been cannulating mouse Wharton’s (submandibular) and Stensen’s (parotid) ducts intraorally and infusing products retrogradelly to the SGs [56], [57]. We have begun to test retrograde infusion of *BM Soup* to SGs of irradiated mice and rats.

Age of the donor reduces the recovery of stem cells in bone marrow and in certain tissues [20], [21], [22], while other studies have reported no significant differences [23], [24], [58]. Our study only compared donor mice aged 8 wks versus 20 wks old while studies such as the one by Wang and colleagues [20] used BM cells from donor mice as old as 2.5 yrs. Our goal, for this study, was to test that the therapeutic effect of *BM Soup* on irradiated SGs is not limited to young mice only, but was also feasible in more matured/older mice. We continue to house C3H donor mice from the same batch/lot as the ones used in the current study and will sacrifice them approximately at 2 yrs of age to test their *BM Soup* efficiency.

There are limitations to this study. First our IR model used a single high dose of 15 Gy. While certainly useful experimentally in mice, this is unlike patients receiving fractionated IR, with 1.5–2.5 Gy/day, 5 days/week, and for up to 6 weeks. Dr Bruce Baum’s group reported that a 15 Gy single-dose scheme and a 5-day × 6 Gy fractionated scheme both yielded severe salivary hypofunction (40–60% salivary flow reduction) [59], [60], [61]. This adapted scheme is more convenient than the human regimen, yet one that leads to significant salivary hypofunction for testing in C3H mice. Future experiments testing *BM Soup* will use this fractionate IR scheme. The second limitation of this study was that *BM Soup* was injected between day 5–7 post-IR, and not in a situation where there was already an established reduction in salivary flow (e.g., at 8 weeks post-IR in the mouse model or 12 months post-IR in humans). Thus the therapeutic effect of *BM Soup* in these situations remains to be tested. The third limitation of this study is it was not aimed at characterizing all the factors/ingredients of *BM Soup* (such as proteins, transcription factors, growth factors, cytokines, etc). The current study was specifically geared at examining a subset of factors known to be involved in SG development/repair/regeneration, in relation to a proposed new treatment, the *BM Soup*. Our gene expression data showed that several growth factors (FGF2, IGF, VEGF, BMP7, EGF, NGF) were up-regulated in *BM Soup*-treated mice (Fig. 4). However, we only examined these mRNA levels at one time point (8 wks post-IR) and thus this gene expression profile may change at subsequent time points. It is possible that the therapeutic effect of *BM Soup* will lessen during a longer follow-up period (such as 16 and 24 week post-IR). Also, we plan to test which “*Cell Soup*” (from subpopulation of cells in BM or from other tissues) can best restore SG function in irradiated mice. Needless to say that this study reports a proof-of-concept of the successful use of *BM Soup* into irradiated SG, and that much more work remains to characterize the components and utilization of *BM Soup*.

## Materials and Methods

### Animals

All procedures with animals were carried out under protocols approved by the Facility Animal Care Committee of McGill University. Female C3H mice of 8 weeks old (Charles River, Montreal, QC, Canada) were used as recipient mice. Donor mice were 8 wks or 20–22 wks old male C3H mice.

### Irradiation (IR)

**Figure 9:**
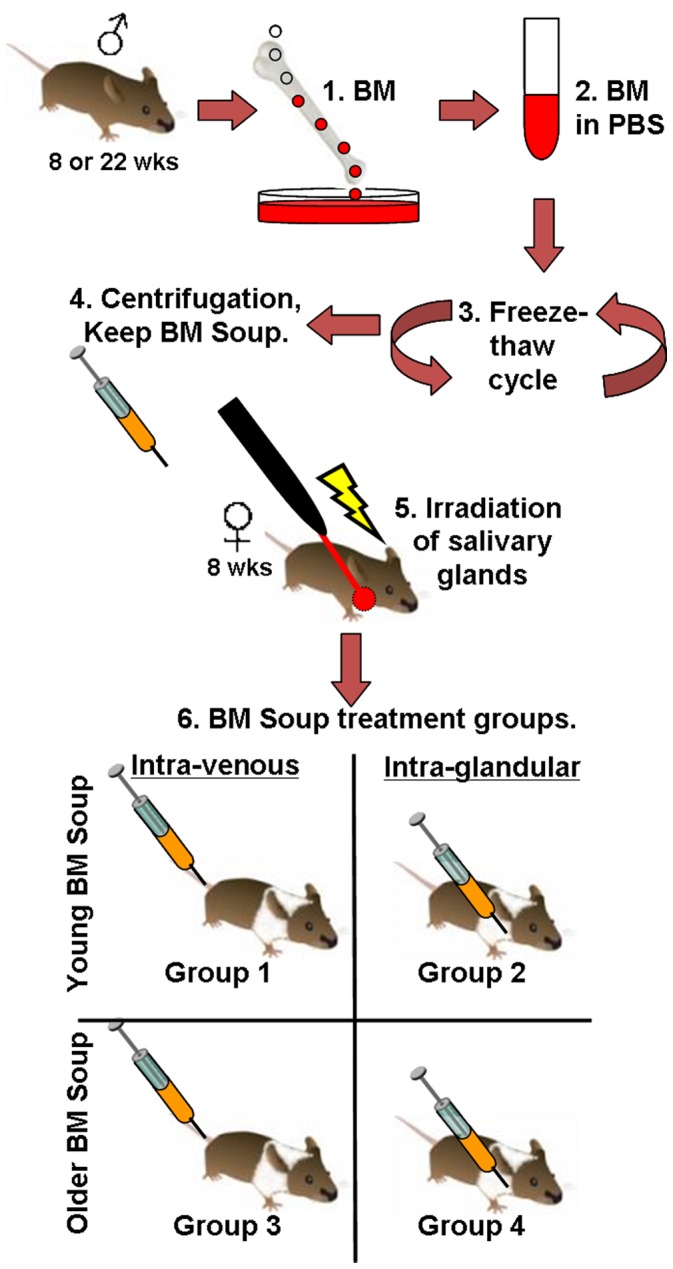
Bone Marrow Soup (BM Soup) preparation and study design. **Step 1.** Bone marrow (BM) is flushed from femur/tibia bones of young (8 wk-old) or older (22 wk-old) male donor mice. **2.** BM cells are suspended in PBS, strained through a filter and washed. **3.** *BM Soup* is prepared by subjecting the BM cells to three freeze-thaw cycles of -80C to +37C degrees. **4.** Microcentrifugation at 13,500 g×30 min, 4C degrees to remove insoluble materials. The supernatant (*BM Soup)* is kept on ice until injections. **5.** Salivary glands of recipient female mice (8 wks old) are locally irradiated with 15 Gy. **6.** Within 5–7 days post-irradiation, *BM Soup* (from young or older donor mice) is injected intra-venously (tail vein) or intra-glandularly (submandibular gland). Mice were monitored for 8 wk post-irradiation.

Mice were anesthetized with 0.3 µl/g body weight of a 60 mg/ml ketamine and 8 mg/ml xylazine (Phoenix Scientific) solution given by intra-muscular injection, and placed in a restraining device. Salivary glands (SGs) of female C3H mice (8-wk old) were damaged by local neck IR of 15 Gy using a Varian Clinac 6EX linear accelerator in a single field anterior-posterior setup (Fig. 9). The radiation field was defined by precise jaw collimation to a slit of 1.2 cm, thereby confining the IR region in the mice to the area of the SGs. The machine output for these specialized narrow fields was measured using a PTW microLion chamber (Type 31018) cross-calibrated against a calibrated secondary standard Farmer-type ionization chamber in a 10×10 cm^2^ reference field size. Additional buildup material was used to ensure a uniform dose as a function of depth throughout the IR region. Typically 5 mice were IR simultaneously and a typical IR took 4.4 min. The IR caused a 40–60% reduction in saliva output at day 5 post-IR. Mice were randomized into 4 different groups (N = 5 to 9 mice per group) and were followed for 8 weeks post-IR. The treatment group (“*IR+BM Soup*”) consisted of IR female mice receiving I.V. injections (tail vein) of *BM Soup* from male donors between day 5–7 post-IR and were compared to IR mice receiving I.V. PBS injections (“*IR+PBS*”, negative control group, vehicle injection group). The positive control group (“*Control*”) was C3H mice with normal SG function which were sedated and placed in the restraining device inside the irradiator but the unit was off (i.e. sham irradiation). Therefore these “*Control*” mice were expected to have normal SG function during the 8 weeks follow-up period. A treatment comparison group (“*IR+whole BM*”) receiving injections of “live whole BM cells” was included in the present study because we have previously shown that these transplants were relatively efficient in re-establishing normal functions to IR-damaged SGs [15], and wanted to compare its therapeutic efficiency against our newly proposed *BM Soup* treatment.

### Bone Marrow (BM) Cells or *BM Soup* Preparation and Transplantation

BM cells were harvested from 8 wk-old male C3H mice. Briefly, BM cells were flushed from tibias and femurs with cold PBS. The suspension was then strained through a 40-µm filter and washed again with PBS (Fig. 9). Cell concentration was adjusted to 10^7^ cells/100 µl PBS. *BM Soup* was prepared as described by Yeghiazarians et al [16], with modifications, by subjecting BM cells to three cycles of freeze-thaw on a container with dry ice placed in a -80C freezer and thawing in a 37C bath, followed by microcentrifugation at 13,500 g at 4C for 30 min to remove insoluble materials. Protein concentration of *BM Soup* was ∼1.3 ug/ul (from 10^7^ cells). *BM Soup* or BM cells were kept on ice until injections in irradiated female recipient C3H mice. The injection of 100 ul containing 1×10^7^ BM cells or *BM Soup* was done via the tail vein, and these transplantations were repeated twice per week during two consecutive weeks. For experiments where *BM Soup* was delivered by intra-glandular injections, an incision in the neck area was performed and the mouse submandibular glands localized. *BM Soup* was injected through the capsule of both submandibular glands. Two injections of 25 ul each/per gland were given. Each delivery route (either IV or intra-glandular) was adjusted to 1×10^7^ cell equivalents of *BM Soup*/mouse. Also each group of female mice receiving *BM Soup* I.V. or intra-glandular was further subdivided into *BM Soup* from a young (8 wks old) versus an older (20–22 wks old) mouse donor. The total number of mice used for this study was 77 mice.

### Measurements of Salivary Function

We performed both quantitative (salivary flow) and qualitative (composition) measurements of saliva (described below).

### Secretory Function of the Salivary Glands (Salivary Flow Rate; SFR)

SFR of mice was obtained by intra-muscular injection of 0.3 µl/g body weight of a 60 mg/ml ketamine and 8 mg/ml xylazine mixture. Whole saliva was collected after stimulation of secretion using 0.5 mg Pilocarpine (Sigma)/kg body weight administered subcutaneously. Saliva was obtained from the oral cavity by micropipette, placed into pre-weighed 0.5 ml microcentrifuge tubes. SFR was determined by volume-weight of saliva/10 min/body weight. SFR was determined at baseline, day 5, and at week 8 post-IR. At time of sacrifice (week 8 post-IR), the mice submandibular glands were harvested and weighed.

### Analysis of Saliva Quality/composition

Concentrations of epidermal growth factor (EGF) in saliva were measured by ELISA method (R&D Systems, Minneapolis, MN, USA) [15]. The total protein concentration of saliva was measured by the bicinchoninic acid assay method (BCA; Thermo Scientific, Pierce, IL, USA). Salivary electrolytes such as sodium, potassium, chloride, and calcium were analyzed on a Beckman Coulter DXc 800 automated chemistry analyzer using the urine chemistry mode (Beckman Coulter Synchron systems, CA) [36].

### Salivary Tissue Analysis

#### 1. Blood vessel/capillary density

Five-micrometer thickness tissue sections were immuno-histochemically stained using the Blood Vessel Staining Kit (Chemicon, MA, USA) with an antibody to von Willebrand Factor (vWF). After deparaffinization and rehydration, tissue sections were treated three times with a 10 mM citrate buffer solution (pH 6.1) in a 600 W microwave and then allowed to cool to room temperature for 30 min before blocking overnight at 4°C. Rabbit anti-mouse vWF was used (1∶100) for 2 hours at room temperature. After that, we followed the manufacturer’s instructions. The percentage of surface area occupied by blood vessels was assessed by light microscopy under 400× magnification using 3 sections for each slide. At least 10 fields per section were accounted using NIH Image J software (NIH, Bethesda, USA).

#### 2. Cell proliferation

Tissue sections were blocked for endogenous peroxidase activity with 3% H_2_O_2_ in methanol for 10 min. Cell proliferation staining was performed with the Zymed PCNA staining kit (Invitrogen, Carlsbad, CA, USA). Before antibody labeling, the slides were treated three times with a citrate buffer solution (mentioned above) in a 600 W microwave for 5 min. Thereafter, slides were processed with routine indirect immunoperoxidase techniques. Two examiners independently counted the absolute number of PCNA positive cells in a blinded manner in five randomly chosen sections (n = 5 glands/group) at the magnification of 400×.

#### 3. Periodic acid–Schiff stain (PAS)

Acinar cells were detected using PAS method (Sigma-Aldrich, St-Louis, MO, USA). The percentage of surface area occupied by acinar cells (reported as a percentage of the salivary tissue surface examined) was assessed by light microscopy under X 400 magnification of 10 representative fields per gland/mouse with NIH Image J software.

#### 4. Immunostaining

Frozen submandibular gland tissue sections (6–8 µm) were obtained from control (sham-irradiated), irradiated, and *BM Soup*-treated C3H mice. The following primary antibodies were used: a) rat anti-sodium-potassium-2 chloride co-transporter, (NKCC1, donated by RJ Turner, NIH) to localize salivary acinar and intercalated ductal cells, b) rat anti-c-Kit (CD117, BD Biosciences, Mississauga, ON, Canada) to localize salivary progenitor cells [31], c) rabbit anti-α-amylase (Sigma-Aldrich) for serous acini cells, d) rabbit anti-α-smooth muscle actin (Abcam, Cambridge, MA, USA) for myoepithelial cells, e) rabbit anti-cytokeratin-5 (CK5, Sigma-Aldrich, Oakville, ON, Canada) for salivary progenitor cells found in basal ductal cells. In addition, a non-immune rat or rabbit isotype control antibody (Zymed Labs) was used as a ‘negative control primary antibody’. Tissue sections were incubated with the respective primary antibody (or its isotype control antibody) overnight at 4°C**.** Tissue sections were then incubated with a secondary antibody for 1 h at room temperature in the dark. Secondary antibodies used were either a donkey anti-rat- fluorescein isothiocyanate-conjugated (FITC), rabbit- Rhodamine Red-X-conjugated (RRX), or rat-RRX (Jackson ImmunoResearch Laboratories, West Grove, PA, USA). Then, 4,6-diamidino-2-phenylindole, dihydrochloride (DAPI; Invitrogen, San Francisco, CA, USA) was added for 3–5 min. All primary and secondary antibodies were diluted (1∶100) in phosphate-buffered saline (PBS) containing 5% donkey serum (Jackson ImmunoResearch Labs). Fluorescence images were taken using a Leica DM4000 fluorescent microscope equipped with Volocity software (PerkinElmer Inc). Images shown are representative of at least 5 separate experiments with multiple images taken per slide. Quantification of the protein fluorescence (i.e. its intensity) was determined by Volocity software. The average fluorescence of the protein expressed within 3–5 fields of 400 um^2^ area/per gland was computed.

#### 5. Fluorescence in situ hybridization (FISH)

Serial frozen tissue sections (8 um thickness) were fixed in a 2% paraformaldehyde fixative for 10 minutes at room temperature. Following, serial dehydration, a digoxigenin-labeled riboprobe was used to detect a repeat sequence (pY353B) on the mouse Y-chromosome. The digoxigenin-labeled probe was applied to the tissue section and hybridized at 81°C for 12 min, then followed by 30 minutes incubation at 55°C. Slides were washed in saline-sodium citrate (SSC) followed by a stringency wash at 40°C. Then, a HRP-conjugated antibody to digoxigenin (1/400, Roche, Indianapolis, IN, USA) was added to detect the riboprobe. The positive Y chromosomal signal was observed as a red color once the high affinity substrate of HRP, Alexa-tyramide-594 reagent (TSA System, Invitrogen) was applied. Finally, all sections were stained with DAPI (Invitrogen) to label all nuclei and mounted with 10 mM Tris (hydroxymethyl) - aminomethane, [pH 7.4]) buffer. The slides were visualized using a Leica DM4000 fluorescent microscope equipped with Volocity software.

#### 6. Quantitative Real-Time PCR (qRT-PCR)

Gene expression analyses were performed using an Applied BioSystems qRT-PCR (model 7500). Total RNA was extracted from the submandibular glands with TRIZOL reagent (Invitrogen, Carlsbad, CA). The first-strand cDNA synthesis was performed by using Thermoscript RT-PCR system (Invitrogen). qRT-PCR was done using 1 µg RNA per sample and TaqMan Universal Master Mix (Applied Biosystems). The probes and primers chosen were for angiogenic growth factors such as VEGF and FGF2 (assay IDs were Mm01281449 and Mm00433287, respectively), resident ductal progenitor cells (CK5, assay ID: Mm00503549), acinar cells (AQP5, assay ID: Mm00437578)**,** transcription factors involved in stem cell renewal or differentiation such as Sox2, Klf4, and Sox10 (assay IDs: Mm00488369, Mm00516104, and Mm01300162), growth factors that impact cell fate decision such as BMP7, EGF, NGF, IGF-1R (assay IDs: Mm00432102, Mm00438696, Mm00443039, and Mm00802841), cell cycle or apoptosis such as Cyclin-1, Caspase-3 and Caspase-8 (assay IDs: Mm00432359, Mm01195085, Mm00802247), bicarbonate anion exchanger **(**SLC4 solute carrier family 4 anion exchanger, assay ID: Mm01347935), and tissue remodeling such as MMP-2 and MMP-9 (assay IDs: Mm00439498 and Mm00442991). Glyceraldehyde-3-phosphate dehydrogenase (GAPDH, assay ID: Mm99999915) was used as an endogenous reference running at 50°C for 2 min, 95°C for 10 min, and 40 cycles [95°C for 15 s, 60°C for 1 min]).

#### 7. Statistical analysis

ANOVA analysis with Tukey’s Post-Hoc test were used to determine statistical differences (P<0.05) between the mouse groups. The statistical program was SPSS version 17 (IBM, USA).
